# Clinical and prognostic significance of preoperative plasma hyperfibrinogenemia in gallbladder cancer patients following surgical resection: a retrospective and *in vitro* study

**DOI:** 10.1186/1471-2407-14-566

**Published:** 2014-08-05

**Authors:** Yi-Jun Shu, Hao Weng, Run-Fa Bao, Xiang-Song Wu, Qian Ding, Yang Cao, Xu-An Wang, Fei Zhang, Shan-Shan Xiang, Huai-Feng Li, Mao-Lan Li, Jia-Sheng Mu, Wen-Guang Wu, Ying-Bin Liu

**Affiliations:** Department of General Surgery and Laboratory of General Surgery, Xinhua Hospital, Affliated with Shanghai Jiao Tong University, School of Medicine, No.1665 Kongjiang Road, Shanghai, 200092 China; Institute of Biliary Tract Disease, Shanghai Jiao Tong University, School of Medicine, No. 1665 Kongjiang Road, Shanghai, 200092 China

**Keywords:** Gallbladder cancer, Coagulation assays, Hyperfibrinogenemia, Prognosis

## Abstract

**Background:**

Coagulation and fibrinolysis activation is frequently observed in cancer patients, and the tumors in these cases are thought to be associated with a higher risk of invasion, metastasis, and worse long-term outcome. The objective of this study was to elucidate the prognostic significance of blood coagulation tests and various clinicopathological characteristics in patients with gallbladder cancer (GBC) after surgical resection.

**Methods:**

We retrospectively reviewed the medical records of 115 patients with histologically confirmed GBC who underwent surgical resection in our department. The prothrombin time (PT), activated partial thromboplastin time (aPTT), thrombin time (TT), international normalized ratio (INR), fibrinogen levels, and platelet counts were measured pretreatment at the time of diagnosis. The predictive value of fibrinogen levels for tumor staging was evaluated using a receiver operating characteristic (ROC) curve analysis. Correlations between the preoperative hyperfibrinogenemia and clinicopathological characteristics were analyzed, and univariate and multivariate survival analyses were performed to identify the factors associated with overall survival (OS). Cancer cell migration and invasion *in vitro* were examined to investigate the function of fibrinogen in GBC cell migration.

**Results:**

The plasma levels for all coagulation tests, with the exception of INR, were significantly different between the GBC patients and control patients (p < 0.001). Hyperfibrinogenemia (>402 mg/dL) was associated with poorly differentiated tumors, advanced tumor invasion, lymphatic metastasis, and advanced tumor stage (p < 0.001), and had a statistically significant adverse effect on survival (p = 0.001). In the multivariate analysis, hyperfibrinogenemia (p = 0.031) was independently associated with worse OS, tumor stage (p = 0.016), margin status (p < 0.001), and lymphatic metastasis (p = 0.035). Moreover, cell migration and invasion *in vitro* were significantly enhanced by fibrinogen.

**Conclusions:**

Preoperative plasma fibrinogen levels was associated with tumor progression and may be an independent marker of poor prognosis in GBC patients. Furthermore, fibrinogen may contribute to cell migration by inducing epithelial-mesenchymal transition.

## Background

Gallbladder cancer (GBC) is the most common biliary tract malignancy and the seventh most common gastrointestinal cancer [1]. The Surveillance, Epidemiology, and End Results (SEER) program estimates the incidence of GBC at 2.5 per 100,000 persons. Despite the relatively low incidence rate, GBC-associated mortality is higher than that of other cancers [2]. The median survival of GBC patients is less than 1 year, which is due to early metastasis via lymphatic, perineural, and hematogenous routes, as well as direct invasion into the liver [3]. Although the tumor, node, and metastasis (TNM) staging system of the American Joint Committee on Cancer (AJCC) is the most widely used system, there is no worldwide consensus on the optimal system or marker for preoperatively predicting the prognosis of patients with GBC.

For more than one century, platelet and blood coagulation abnormalities have been described in cases of malignancy [4, 5], and several mechanisms have been proposed linking tumor biology to coagulation [6]. For example, patients with tumors in the lung, pancreas, and gastrointestinal tract are thought to be more prone to developing a hypercoagulable state [7].

Rather than serving as a mere trigger for increased thromboembolic events, cancer-induced hemostatic activity has been shown to promote tumor growth and cancer cell dissemination [8]. Both coagulation assays and high levels of circulating biomarkers, indicative of coagulation and fibrinolysis activation, have been associated with decreased survival in several tumor types [9–11]. Although previous studies evaluating the effect of combined anticoagulant treatment and chemotherapy in malignancy patients have observed survival benefits, sufficient evidence of such an advantage remains to be demonstrated. Nevertheless, understanding the potential pathways responsible for activated hemostatic and fibrinolytic activity may help identify surrogate markers for novel therapeutic targets.

Therefore, based on our previous basic study [12], the purpose of this study was to assess whether coagulation abnormalities are more frequently encountered in GBC and to delineate the correlation between coagulation function and other clinical variables. A long term study investigating the association between preoperative plasma fibrinogen levels and GBC patient survival has not yet been reported. In this current study, we evaluated the clinicopathological significance of hyperfibrinogenemia and its prognostic relevance for patients with GBC. Finally, we evaluated the relationship between fibrinogen and the migration of GBC cells *in vitro.*

## Methods

### Ethics

Written informed consent for surgical treatment and pathological examination was obtained from all patients according to institutional guidelines. All studies were approved by the Committee for Ethics of Xinhua Hospital, Shanghai Jiao Tong University School of Medicine.

### General information

Between January 2010 and December 2013, 115 patients with histologically confirmed GBC underwent surgical resection at both our department and the sub-unit in Chongming, China. The median age at diagnosis was 67 years, the age range was 38–91 years, and females comprised the majority of the group (n = 78, 67.8%). A total 88 patients (76.5%) had associated biliary tract pathology, including gallstones in 76 patients and gallbladder polyps in 12 patients. Tumors that were histologically diagnosed after cholecystectomy were termed incidental GBC. In our study, the diagnosis of GBC in 12 patients (10.4%) was missed at the time of routine cholecystectomy for gallstones. Well- or moderately differentiated adenocarcinoma was diagnosed in 64.4% of the tumors, and poorly differentiated adenocarcinoma was diagnosed in the other 35.7%. The final disease staging and histological grading was based on the 7th edition of the AJCC manual [13]. Most of the lesions (74.8%) were categorized as AJCC stage II, III, or IV at the time of diagnosis and treatment. Lymph node metastasis (42 cases) was recognized pathologically in 42.6% of the cases, and R0 resections were considered for resection with curative intent [14].

We excluded patients who had a history of malignancy or other simultaneous cancer, had undergone emergency surgery, had a history of heart attack or stroke, and were currently using anticoagulants, corticosteroids, estrogen, or aspirin, which may affect the hemostatic system. Additionally, 50 age- and sex-matched patients with benign disease (cholecystitis) were included in the analysis as a control group.

### Biochemical assays

Venous blood samples were collected preoperatively into tubes containing sodium citrate, immediately centrifuged, and evaluated within 2 h. The prothrombin time (PT), activated partial thromboplastin time (aPTT), thrombin time (TT), international normalized ratio (INR), and fibrinogen were measured with commercially available reagents for the kinetic nephelometric detection system using Diagon Dia-Timer 4 (Diagon Ltd, Budapest, Hungary).

### Surgical strategy and patient follow-up

The surgical protocol for GBC at our center was as follows. For T1 lesions, curative resection was achieved by simple cholecystectomy; for T2-T4 lesions, radical surgery was performed with curative intent, comprising a liver wedge with a 2-cm margin around the gallbladder and an enbloc GB resection with skeletonization of the hepatoduodenal ligament (lymph nodes along the hepatoduodenal ligament and common hepatic artery, and behind the pancreatic head). For these procedures, the “curettage and aspiration dissection technique” was performed using Peng’s multifunctional operative dissector [15]. In cases where the GBC infiltrated the surrounding structures, including the common bile duct, omentum, and colon, combined resection of the invaded organ was performed to achieve a negative margin. For palliative surgery, a biliary T-tube drainage with GBC resection only was conducted in patients with distant metastasis, cachexia, or extensive lymph nodal involvement.

Patients were followed postoperatively every 3 months for 2 years and then every 6 months thereafter. The date of surgery marked the beginning of the follow-up period, which ended at the last follow-up visit (December 2013) or death.

### Cell lines and culture

The human gallbladder cancer cell lines GBC-SD and NOZ were purchased from the Shanghai Cell Institute Country Cell Bank. GBC-SD cells were cultured in high-glucose Dulbecco’s modified eagle’s medium (DMEM) (Gibco, California, USA), and NOZ cells were cultured in William’s medium (Gibco) supplemented with 10% fetal bovine serum (Gibco), 100 μg/mL streptomycin, and 100 U/mL penicillin (Hyclone, Logan, UT) at 37°C in an atmosphere containing 5.0% CO_2_. Cells were routinely grown in 100-mm plastic tissue culture dishes (Corning, New York, USA) and harvested using a trypsin-EDTA solution when they reached the logarithmic growth phase. Cells were maintained in these culture conditions for all experiments.

### Cell migration and invasion assays

For the *in vitro* wound-healing assay, a cell-free area of the culture medium was wounded by scratching with a 200-μL pipette tip. Cell migration into the wound area was monitored in a serum-free medium and photographed using a fluorescence microscope at 0, 24, and 48 h. Then, the effects of fibrinogen (Sigma, St. Louis, MO) on cell migration and invasion were determined using 8-μm transwell filters (BD Biosciences, Franklin Lakes, NJ, USA) with or without Matrigel (BD). GBC-SD (3 × 10^4^) and NOZ (4 × 10^4^) cells in 0.5 μL of serum-free DMEM and William’s media were added to the upper chamber, which contained a non-coated/Matrigel-coated membrane. The same protocol was performed on the treatment group (40 μg/mL fibrinogen). The lower chamber was filled with 500 μL of basal medium comprising 10% fetal bovine serum. After a 24-h incubation at 37°C in a 5% CO_2_ humidified incubator, cells that migrated to the lower compartment were fixed with methanol and stained with crystal violet. Migrated or invaded cells were counted in five randomly chosen fields in each well, and imaging and cell counting were performed at 10× magnification using a fluorescence microscope. The experiments were performed in triplicate.

### Western blot analysis

Cells (1 × 10^7^) were seeded into a cell culture dish and treated with fibrinogen (20 or 40 μg/mL) for 48 h. Cellular proteins were extracted using lysis buffer (Beyotime, Shanghai, China) from the control and treated cells. For western blot analysis, the proteins were separated by SDS-PAGE and blotted onto PVDF membranes. The membrane was blocked in blocking buffer (5% non-fat dry milk) for 1 h at room temperature and incubated with primary antibodies in blocking buffer overnight at 4°C. Anti-E-cadherin and anti-vimentin (both at 1:500, Cell Signaling Technology, Danvers, USA) were used as primary antibodies. β-Actin (Beyotime) was used as a loading control. The blot was then incubated with the appropriate secondary antibody, detected with 10 mL of AP buffer at room temperature for 10–20 min, and photographed. The optical densities of the bands were scanned and quantified using the Gel Doc 2000 (BioRad, Hercules, USA).

### Quantitative real-time PCR (qRT-PCR)

Total RNA was extracted from control and treated cells using Trizol reagent (Takara, Shiga, Japan), and the first-strand cDNA was synthesized from 2 μg of total RNA using random primers and the M-MLV Reverse Transcriptase (Invitrogen, Carlsbad, CA). RNA expression was measured by qRT-PCR using the SYBR-Green method (Takara) according to the manufacturer’s instructions. Primer sequences were as follows: E-cadherin, forward primer 5′-TGCCCAGAAAATGAAAAAGG-3′, reverse primer 5′-GTGTAYGTGGCAATGCGTTC-3′; vimentin, forward primer 5′-GAGAACTTTGCCGTTGAAGC-3′, reverse primer 5′-GCTTCCTGTAGGTGGCAATC-3′; GADPH, forward primer 5′-GAGAGACCCTCACTGCTG-3′, reverse primer 5′-GASTGGTAGATGACAAGGTGC-3′. Differences in expression were assessed by 2^-ΔΔCt^ relative quantitative analysis.

### Statistical analysis

Continuous variables are presented as the mean (standard deviation), and categorical variables are presented as the frequency and proportion (%). The relationships between coagulation tests, including PT, aPTT, TT, INR, fibrinogen, and clinicopathological characteristics, were evaluated and compared using the unpaired *t*-test and one-way analysis of variance. The predictive performance of fibrinogen levels for tumor staging was evaluated using a receiver operating characteristic (ROC) curve analysis. The positive predictive value (PPV) was calculated using a cut-off value selected from the ROC curve. The association between hyperfibrinogenemia and various clinicopathological factors was assessed using Fisher’s exact test. The 1- and 3-year survival rates were calculated using Kaplan-Meier analysis and compared using the log-rank test. Variables with p-values < 0.05 on the univariate analysis were included in the final multivariate survival analysis that was conducted using the Cox proportional hazards model. Statistical significance was designated at p < 0.05. Statistical analysis was performed using SPSS 19.0 software.

## Results

### Comparison of coagulation tests between GBC patients and cholecystitis controls

The plasma levels for all coagulation tests, including PT, aPTT, TT, fibrinogen, and platelet counts, were significantly different between the GBC patients and cholecystitis controls (p < 0.05, Table 1). This result suggested a higher tendency for an activated coagulation cascade in patients with malignancy compared with those with benign inflammation.

**Table 1:** **The serum coagulation test results in patients with GBC and cholecystitis**

Coagulation tests	Patients (n = 115)	Controls (n = 50)	p
	Mean (standard deviation)	Mean (standard deviation)	
PT (sec)	***11.6 (1.3)***	***10.5 (1.1)***	***<0.001***
aPTT (sec)	***27.8 (4.7)***	***32.2 (4.3)***	***<0.001***
TT (sec)	***15.4 (3.4)***	***13.8 (1.5)***	***0.002***
INR	1.1 (0.9)	1.0 (0.1)	0.325
Fibrinogen (mg/dL)	***407 (121)***	***311 (67)***	***<0.001***
Platelets (×10^4^/mm^3^)	***221.7 (73.4)***	***188.2 (55.3)***	***0.013***

Bold italics indicate statistically significant values (p < 0.05).

### Correlations between coagulation tests and clinical variables

The relationships between coagulation variables and tumor progression, including histologic differentiation, tumor invasion, lymph node metastasis, TNM stage, and clinical characteristics of the patients, are summarized in Table 2. A statistically significant association was observed between the preoperative plasma fibrinogen levels and tumor differentiation of GBC; specifically, GBC patients showing poor differentiation exhibited higher plasma fibrinogen levels compared with patients with moderate and well-differentiated tumors (p = 0.002). More severe GBC stages, including tumor invasion, lymph node metastasis, and TNM stage, were also associated with higher fibrinogen levels compared with limited-stage disease (tumor invasion, p = 0.016; LN metastasis, p = 0.005; and TNM stage, p < 0.001).

**Table 2:** **Correlation between coagulation variables and clinicopathological characteristics in GBC patients**

Variable	N	Coagulation tests					
		PT (s)	aPTT (s)	TT (s)	INR	Fibrinogen (mg/dL)	PLT (×10 ^4^/mm ^3^)
Gender							
Male	37 (32.2%)	11.7 (1.6)	***29.2 (5.1)***	14.5 (3.0)	1.3 (1.5)	423.7 (127.3)	212.0 (79.6)
Female	78 (67.8%)	11.6 (1.2)	***27.2 (4.4)***	15.8 (3.6)	1.0 (0.1)	398.2 (118.0)	226.3 (69.6)
		(p = 0.642)	***(p = 0.028)***	(p = 0.126)	(p = 0.323)	(p = 0.293)	(p = 0.330)
Age (years)							
<60	29 (25.2%)	11.3 (1.2)	28.2 (5.0)	14.2 (2.9)	1.0 (1.0)	373.7 (136.2)	223.6 (72.9)
≥60	86 (74.8%)	11.7 (1.4)	27.7 (4.6)	15.8 (3.5)	1.1 (1.0)	417.5 (114.3)	221.1 (73.4)
		(p = 0.123)	(p = 0.650)	(p = 0.651)	(p = 0.490)	(p = 0.653)	(p = 0.874)
Histologic differentiation							
Well	15 (13.0%)	11.4 (1.0)	28.0 (4.6)	16.4 (3.3)	1.6 (0.9)	***357.1 (86.4)***	214.7 (61.6)
Moderate	59 (51.3%)	11.5 (1.3)	27.5 (5.1)	15.0 (2.7)	1.0 (0.1)	***381.5 (116.3)***	214.9 (74.1)
Poor	40 (34.8%)	11.7 (1.4)	28.0 (4.1)	15.3 (4.2)	1.1 (0.1)	***454.3 (116.6)***	231.0 (73.5)
Undifferentiated	1 (0.9%)						
		(p = 0.593)	(p = 0.842)	(p = 0.480)	(p = 0.060)*	***(p = 0.002)*** ^**^	(p = 0.526)
Tumor invasion							
Tis-T_1_	18 (0.2%)	11.9 (1.5)	28.7 (5.1)	***17.0 (3.2)***	1.0 (0.1)	***349.9 (68.7)***	210.6 (63.9)
T_2_-T_4_	95 (0.8%)	11.5 (1.3)	27.8 (4.7)	***14.9 (3.4)***	1.1 (1.0)	***421.9 (127.1)***	223.1 (75.0)
Not available	2 (0.0%)						
		(p = 0.262)	(p = 0.423)	***(p = 0.038)***	(p = 0.643)	***(p = 0.016)***	(p = 0.509)
Lymph node metastasis							
Absent	73 (63.5%)	11.6 (1.3)	27.8 (4.8)	15.7 (3.2)	1.2 (1.1)	***382.7 (116.0)***	***208.5 (64.5)***
Present	42 (36.5%)	11.5 (1.5)	27.9 (4.6)	14.7 (3.8)	1.0 (0.1)	***447.7 (120.0)***	***244.7 (81.4)***
		(p = 0.759)	(p = 0.917)	(p = 0.223)	(p = 0.524)	***(p = 0.005)***	***(p = 0.010)***
TNM stage							
0-I	22 (19.1%)	11.9 (1.3)	27.7 (5.1)	16.9 (3.2)	1.0 (0.1)	***331.4 (86.3)***	208.6 (67.0)
II-IV	93 (80.9%)	11.5 (1.3)	27.9 (4.6)	14.9 (3.4)	1.1 (1.0)	***432.9 (120.9)***	224.8 (74.3)
		(p = 0.260)	(p = 0.891)	(p = 0.057)	(p = 0.616)	***(p < 0.001)***	(p = 0.353)

GBC, gallbladder carcinoma; PT, prothrombin time; aPTT, activated partial prothromboplastin time; TT, thrombin time; INR, international normalized ratio; PLT, platelets.

Bold italics indicate statistically significant values (p < 0.05).

*For Well vs. Moderate, p = 0.021; Moderate vs. Poor, p = 0.870; Well vs. Poor, p = 0.036.

**For Well vs. Moderate, p = 0.458; Moderate vs. Poor, p = 0.002; Well vs. Poor, p = 0.005.

### Diagnostic performance of preoperative plasma fibrinogen levels for tumor staging in GBC patients

**Figure 1:**
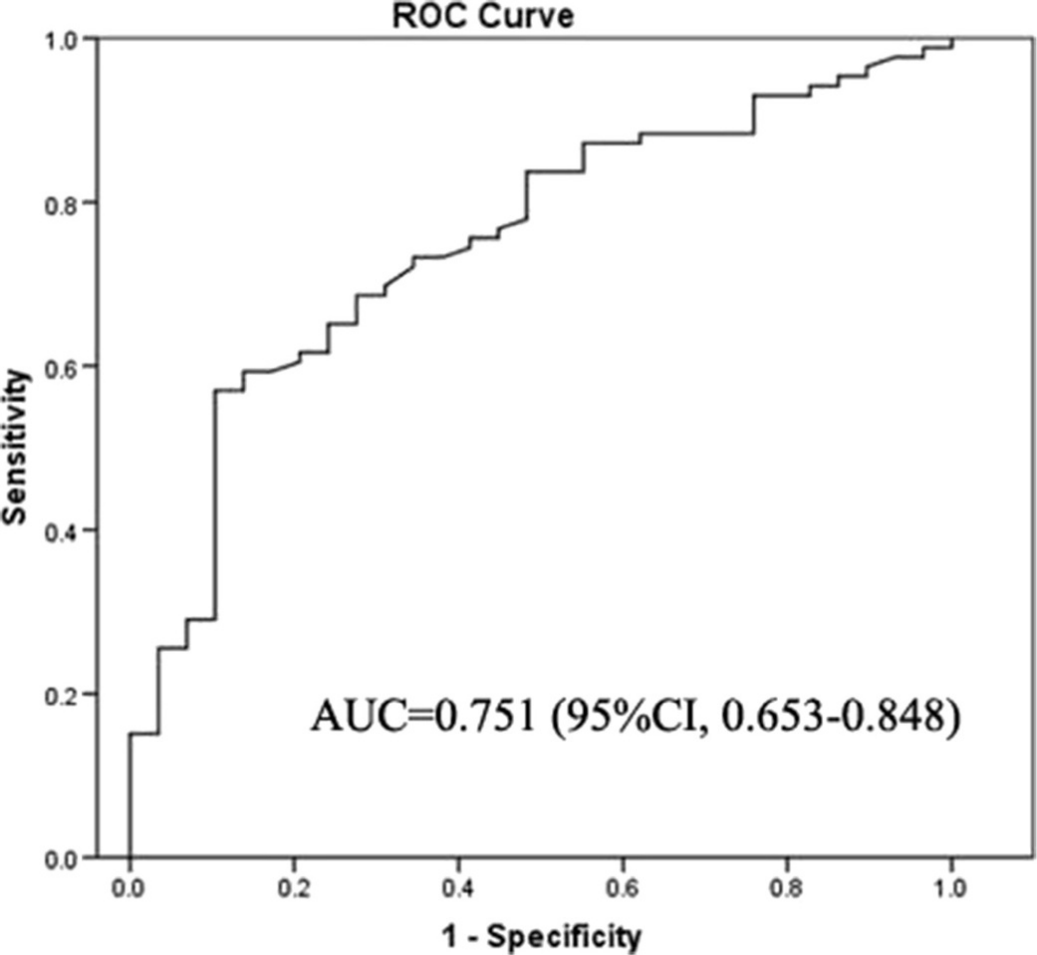
**Receiver operating characteristic (ROC) curve analysis to predict GBC stage.** The area under the ROC curve (AUC) indicates the diagnostic power of preoperative plasma fibrinogen levels.

With the fibrinogen levels correlating with TNM stage, the diagnostic performance of fibrinogen for tumor staging in patients with GBC was further investigated. We found that the area under the ROC curve (AUC) of the fibrinogen levels was 0.751 (95% CI: 0.65–0.85, Figure 1).

The optimal cut-off value for the fibrinogen concentration (402 mg/dL) was selected based on an ROC curve analysis; thus, plasma fibrinogen levels >402 mg/dL were defined as hyperfibrinogenemia. At a 402 mg/dL cut-off, the fibrinogen concentration had a PPV of 92.73%, suggesting that hyperfibrinogenemia may be a valuable biomarker for predicting advanced GBC.

### Correlations between preoperative hyperfibrinogenemia and clinicopathological characteristics

Because the frequency of hyperfibrinogenemia was much higher in patients with malignancy (18.3% in cholecystitis controls vs. 47.8% in GBC patients; *χ*^2^ = 14.60, p < 0.001), we grouped the clinicopathological factors of the 115 GBC patients according to the presence or absence of preoperative hyperfibrinogenemia. As shown in Table 3, preoperative hyperfibrinogenemia was significantly correlated with histological differentiation (p < 0.001), tumor invasion (p = 0.019), nodal metastasis (p < 0.001), and TNM stage (p < 0.001).

**Table 3:** **Demographic characteristics of GBC patients with and without hyperfibrinogenemia**

Variables	Plasma fibrinogen levels (mg/dL)		p ^a^
	≤402 (n = 60 52.2%)	>402 (n = 55 47.8%)	
Gender			
Male	***14 (12.2%)***	***23 (20%)***	***0.034***
Female	***46 (40%)***	***32 (27.8%)***	
Age			
<60 years	19 (16.5%)	10 (8.7%)	0.096
≥60 years	41 (35.7%)	45 (39.1%)	
Incidental GBC	16 (13.9%)	9 (7.8%)	0.181
Associated gallstone			
Absent	18 (15.7%)	21 (18.3%)	0.355
Present	42 (36.5%)	34 (29.6%)	
Liver function			
ALT	63.9 ± 160.8	89.0 ± 148.5	0.439
AST	48.3 ± 99.8	63.9 ± 106.0	0.471
Serum total bilirubin (mg/dL)	23.7 ± 44.4	54.3 ± 95.1	0.056
Albumin (g/dL)	***39.5 ± 4.6***	***37.4 ± 4.9***	***0.04***
Hemoglobin (g/dL)	128.7 ± 15.3	122.7 ± 19.5	0.087
Platelet count (×10^4^/mm^3^)	209.3 ± 63.1	235.3 ± 80.7	0.056
WBC count (×10^9^/mm^3^)	***6.85 ± 3.84***	***8.30 ± 3.45***	***0.048***
CA19-9 (U/mL)	131.6 ± 263.7	474.1 ± 370.9	0.132
CEA (ng/mL)	6.3 ± 21.7	13.4 ± 47.6	0.358
CA125 (U/mL)	***35.3 ± 55.8***	***95.1 ± 141.6***	***0.025***
Histologic differentiation			***<0.001***
Well	***11 (9.6%)***	***4 (3.5%)***	
Moderate	***38 (33.0%)***	***21 (18.3%)***	
Poor	***11 (9.6%)***	***29 (25.2%)***	
Undifferentiated	***1 (0.9%)***		
Tumor invasion			
Tis-T_1_	***15 (13.0%)***	***5 (4.4%)***	***0.019***
T_2_-T_4_	***42 (36.5%)***	***49 (42.6%)***	
Not available	***3 (2.6%)***	***1 (0.9%)***	
Lymph node metastasis			
Absent	***48 (41.7%)***	***25 (21.7%)***	***<0.001***
Present	***12 (10.4%)***	***30 (26.1%)***	
TNM stage			
0-I	***25 (21.7%)***	***4 (3.5%)***	***<0.001***
II-IV	***35 (30.4%)***	***51 (44.4%)***	

Bold italics indicate statistically significant values (p < 0.05).

GBC, gallbladder carcinoma; ALT, alanine aminotransferase; AST, aspartate aminotransferase; WBC, white blood cell count; CA, carbohydrate antigen; CEA, carcinoembryonic antigen; TNM, tumor, node, metastasis classification system; CBD, common bile duct.

^a^ *χ* ^2^ test or Student's *t* test.

### Survival analysis

The median follow-up time was 16 months (range, 1–36 months). Six patients (5.2%) were lost to follow-up, and the median survival time for all patients was 12.5 months (95% CI, 10.44–14.49 months). During the follow-up period, 79 patients (68.7%) died owing to disease-related factors.

**Figure 2:**
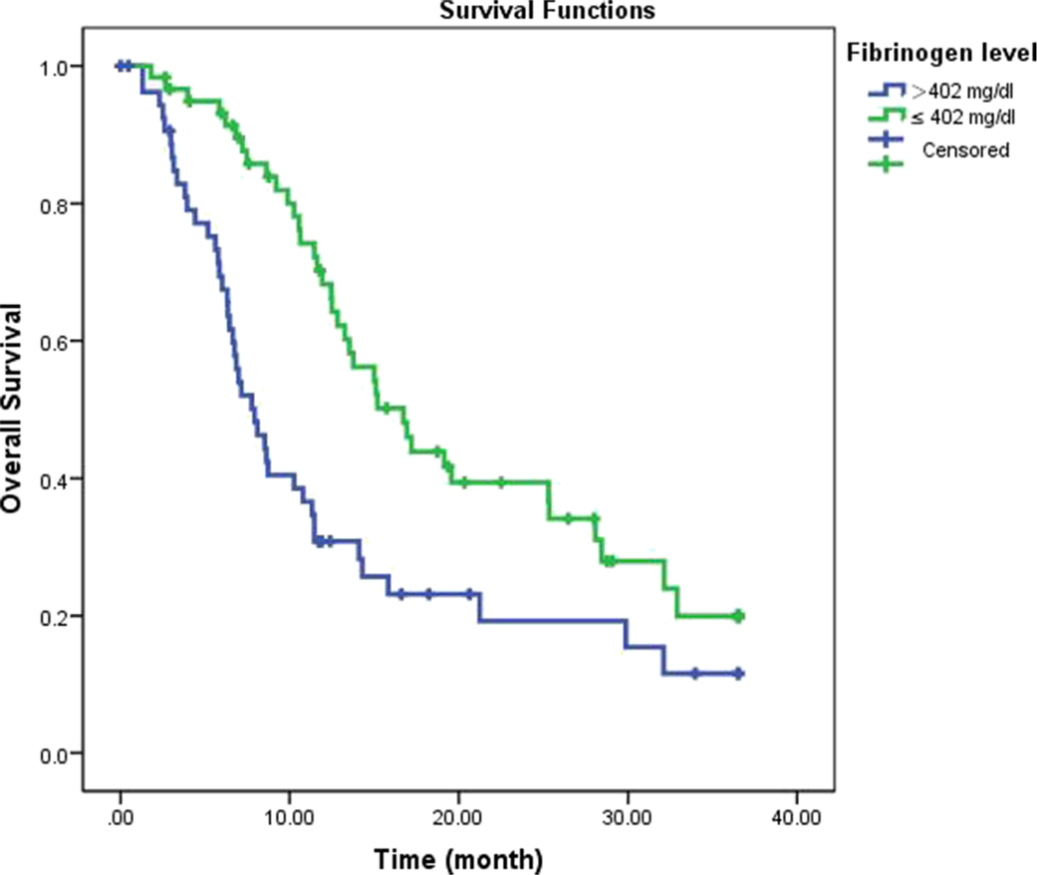
**Survival curve according to the presence of preoperative hyperfibrinogenemia.** Data compares hyperfibrinogenemia vs. non-hyperfibrinogenemia patients (p = 0.001).

Evaluation of the effect of clinical variables confirmed the negative impact of advanced tumor stage, including poor differentiation, deeper tumor invasion, presence of lymph node metastasis, and TNM stage II-IV, and surgery assessment, including R1 resection, absence of lymphadenectomy, (lymphadenectomy p = 0.044; all other variables p < 0.001; Table 4). Among the laboratory variables examined, low hemoglobin and elevated leukocyte concentrations were associated with significantly worse outcomes when compared with concentrations below the median range (p = 0.012 and p = 0.035, respectively). Moreover, the survival of patients with preoperative hyperfibrinogenemia was significantly worse than that of patients without hyperfibrinogenemia (p = 0.001, Figure 2). The actual 1- and 3-year survival rates for patients with hyperfibrinogenemia were 30.8 and 11.6%, respectively, whereas these rates were 68.2 and 34.1%, respectively, for patients without preoperative hyperfibrinogenemia.

**Table 4:** **Univariate analysis of overall survival in GBC patients**

Variables	Characteristics	n	Median OS (months)	HR (95% CI)	p value
Gender	Male	37	14	1.23 (0.76, 2.01)	0.392
	Female	78	11		
Age	≤60 years	29	16	0.66 (0.38, 1.13)	0.122
	>60 years	86	11		
Associated gallstone	Absent	39	13	0.68 (0.43, 1.09)	0.105
	Present	76	12		
ALT	≤75 U/L	85	12	1.35 (0.75, 2.41)	0.315
	>75 U/L	32	10		
AST	≤38 U/L	78	13	1.24 (0.72, 2.13)	0.437
	>38 U/L	37	11		
Serum total bilirubin	≤12 mg/dL	60	14	1.50 (0.91, 2.45)	0.107
	>12 mg/dL	55	10		
Albumin	<35 g/dL	29	11	0.82 (0.45, 1.48)	0.506
	≥35 g/dL	86	12		
Hemoglobin	≤125 g/dL	***53***	***10***	***0.54 (0.34, 0.88)***	***0.012***
	>125 g/dL	***49***	***15***		
WBC count	≤6.8 × 10^3^/mm^3^	***57***	***15***	***1.65 (1.03, 2.64)***	***0.035***
	>6.8 × 10^3^/mm^3^	***45***	***9***		
Fibrinogen	≤402 mg/dL	***60***	***17***	***0.48 (0.31, 0.75)***	***0.001***
	>402 mg/dL	***55***	***8***		
Platelet count	≤215 × 10^4^/mm^3^	58	13	1.44 (0.92, 2.24)	0.108
	>215 × 10^4^/mm^3^	57	11		
CA19-9	≤35 U/mL	54	13	1.36 (0.82, 2.25)	0.239
	>35 U/mL	61	10		
CEA	≤10 ng/mL	62	13	1.61 (0.94, 2.76)	0.078
	>10 ng/mL	53	11		
CA125	≤35 U/mL	66	12	1.60 (0.89, 2.88)	0.11
	>35 U/mL	49	11		
Histologic differentiation	Well	***15***	***32***	***0.51 (0.36, 0.72)***	***<0.001***
	Moderate	***59***	***13***		
	Poor and undifferentiated	***41***	***8***		
Tumor invasion	Tis and T_1_	***20***	***32***	***4.58 (2.15, 9.76)***	***<0.001***
	T_2_, T_3_ and T_4_	***91***	***11***		
Lymph node metastasis	Absent	***73***	***17***	***4.08 (2.49, 6.67)***	***<0.001***
	Present	***42***	***7***		
TNM stage	0 and I	***29***	***33***	***5.97 (2.78, 12.82)***	***<0.001***
	II and IV	***86***	***11***		
Margin status	R0	***69***	***20***	***0.14 (0.09, 0.24)***	***<0.001***
	R1	***46***	***6***		
Combined hepatectomy	No	51	11	0.94 (0.59, 1.49)	0.792
	Yes	54	12		
Combined CBD resection	No	***87***	***14***	***2.06 (1.15, 3.69)***	***0.013***
	Yes	***18***	***9***		
Lymphadenectomy	No	15	8	***1.89 (1.01, 3.56)***	***0.044***
	Yes	83	12		

GBC, gallbladder carcinoma; OS, overall survival; HR, hazard ratio; CI, confidence interval; ALT, alanine aminotransferase; AST, aspartate aminotransferase; WBC, white blood cell count; CA, carbohydrate antigen; CEA, carcinoembryonic antigen; TNM, tumor, node, metastasis classification system; CBD, common bile duct.

Bold italics indicate statistically significant values (p < 0.05).

Multivariate analyses were performed using Cox’s proportional hazards regression. Prognostically significant variables (p < 0.05), including plasma hemoglobin, fibrinogen levels, leukocyte counts, histologic differentiation, tumor invasion, lymph node metastasis, TNM stage, margin status, and lymphadenectomy, were included in the analysis. The results indicated that only stages II-IV, lymph node metastasis, preoperative hyperfibrinogenemia, and R1 resection were independently associated with decreased survival (Table 5).

**Table 5:** **Multivariate analysis for overall survival in GBC patients**

Variables	OS HR (95% CI)	p value
TNM stage	17.42 (1.71, 177.56)	0.016
0-I vs. II-IV		
Lymph node metastasis	2.15 (1.06, 4.40)	0.035
Absent and not available vs. Present		
Fibrinogen	0.52 (0.29, 0.94)	0.031
≤402 mg/dL vs. >402 mg/dL		
Margin status	0.15 (0.07, 0.31)	<0.001
R0 vs. R1		

GBC, gallbladder carcinoma; OS. overall survival; HR, hazard ratio; CI, confidence interval.

### Fibrinogen stimulation of GBC metastasis and invasion *in vitro*

**Figure 3:**
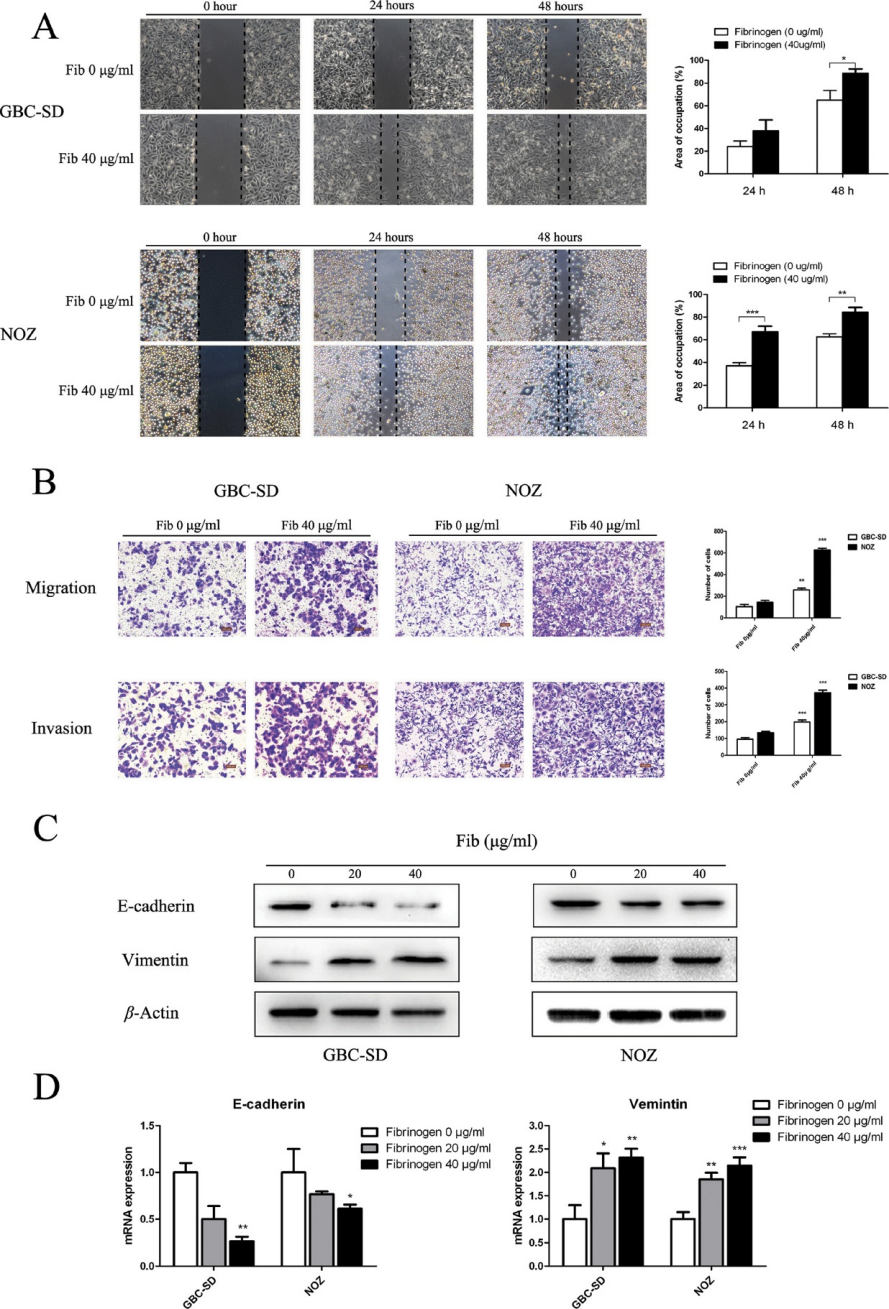
**Fibrinogen stimulation of GBC metastasis and invasion** ***in vitro*** **. (A)** The effect of fibrinogen on the migration of GBC-SD and NOZ cells. The area of the GBC-SD and NOZ cell monolayers treated with fibrinogen (40 μg/mL) increased 24 and 48 h after scratching the media. The histogram shows that fibrinogen significantly improved the migration of GBC-SD and NOZ cells. **(B)** Transwell migration and invasion assays were performed after treating the GBC-SD and NOZ cell lines, with fibrinogen (40 μg/mL), which is summarized in the histograms. **(C and D)** Biomarkers for EMT: E-cadherin and vimentin were analyzed by western blotting and qRT-PCR. All graphs indicate the mean ± SD. Statistical significance as follows: *p < 0.05, **p < 0.01, and ***p < 0.001.

Based on the above results, we concluded that fibrinogen is closely associated with GBC invasion and metastasis. To further explore the role of fibrinogen in the GBC cell lines, we obtained fibrinogen from human plasma. As shown in Figure 3A and B, the wounding-healing and transwell migration assays revealed enhanced migration and invasion of GBC-SD and NOZ cells in the presence of fibrinogen, suggesting that fibrinogen from human plasma may significantly promote the metastasis of GBC cells *in vitro*. Moreover, both western blot analysis and qRT-PCR showed that fibrinogen increased the expression of the mesenchymal marker vimentin, but the epithelial marker E-cadherin expression decreased (Figure 3C and D), indicating that EMT signaling may contribute to the enhanced migration caused by hyperfibrinogenemia. Collectively, our data demonstrate that fibrinogen plays a role in promoting cell metastasis *in vitro*.

## Discussion

Systematic activation of the clotting system has been observed in cancer patients, typically reflected in subclinical abnormalities on conventional coagulation tests [16, 17]. Despite numerous studies investigating the causes of hypercoagulability and thromboembolic complications in patients suffering from malignancy, the responsible mechanisms remain poorly understood [18]. Some evidence suggests that activation of the coagulation and fibrinolytic systems by neoplastic cells facilitates their invasiveness and metastases [19, 20]. Thus, the extent of such activation has been associated with tumor stage and prognosis in some malignancies, such as breast, colorectal, and lung cancer [9–11]. Therefore, markers of clotting may preoperatively predict tumor progression and prognosis in GBC patients.

Various markers indicate an activated hemostatic system, such as thrombocytosis, hyperfibrinogenemia, and elevated D-dimer levels, and have been demonstrated in several cancer types, including gastric, colon, and pancreatic cancer [21–23]. Indeed, the current study also observed a significant difference between the cholecystitis control subjects and GBC patients in coagulation test findings, namely the PT, aPTT, TT, fibrinogen levels, and platelet counts, due to activation of the coagulation and fibrinolytic systems. Furthermore, in our GBC patient subgroup, advanced disease was associated with higher levels of fibrinogen.

Fibrinogen is an essential hemostatic factor that is converted to fibrin, a final product of the hemostatic pathway, by activated thrombin. Fibrinogen is synthesized in the liver and secreted into the circulation, and the levels of this acute-phase reactant increase in response to most forms of tissue injury, infection, or inflammation [24, 25]. Recently, it was reported that the plasma fibrinogen levels was correlated with tumor size, depth of tumor invasion, and metastasis in patients with gastric cancer [26]. Moreover, other studies revealed that lymphatic and hematogenous metastases were greatly reduced in fibrinogen-deficient mice, which indicates a positive role for fibrinogen in the metastatic progression of cancer [27, 28].

Surgical resection is the only effective treatment for GBC [29]. Thus, surgeons require precise preoperative assessment and tumor staging to correctly identify surgical options. Because of its nonspecific symptoms and highly invasive nature, GBC is often only detected when it has reached an advanced stage [30]. In the present study, we observed a positive relationship between hyperfibrinogenemia and TNM stage. In particular, hyperfibrinogenemia predicted tumor staging at a positive predictive rate of 92.7%, indicating that hyperfibrinogenemia can be used as a preoperative indicator of advanced disease in GBC patients. Additionally, the majority of patients with early stage GBC have nonspecific symptoms or vague complaints that are easily confused with symptoms of benign gallbladder disease such as cholelithiasis. In some cases, the diagnosis of GBC has been missed at the time of routine cholecystectomy for benign disease. Stage T_1b_ and T_2_ cases are often misdiagnosed intraoperatively, and these patients must undergo additional surgery to improve their survival. Based on our findings, we suggest that surgeons carefully observe the pathological lesion during surgery and perform a cryosection evaluation in gallbladder disease patients with preoperative hyperfibrinogenemia. Then, the surgical procedures should be suspended until the pathological diagnosis is established to prevent unnecessary surgical trauma to the patient.

Many recent studies have demonstrated that hyperfibrinogenemia, as a marker of coagulation and fibrinolytic activation, is a strong predictor of poor prognosis for gastrointestinal malignancy [22, 23, 26, 31]. Using univariate analysis, our study revealed the significance of hyperfibrinogenemia, and multivariate analysis of survival emphasized the impact of hyperfibrinogenemia on GBC patient survival. In particular, patients with advanced stage disease tended to have decreased survival.

Although fibrinogen synthesis is significantly upregulated by inflammation [32], the molecular mechanisms responsible for changes in GBC metastasis and invasion have not been clearly elucidated. Thus, the present study sought to determine whether fibrinogen levels and GBC development have a cause-and-effect relationship, and whether hyperfibrinogenemia could therefore promote tumor development. In the present study, we showed that co-culture with highly concentrated fibrinogen increased GBC cell migration, invasion, and metastatic capacity, and induced EMT by increasing the expression of vimentin (a mesenchymal marker) and reducing expression of E-cadherin (an epithelial marker). EMT plays a central role in tumor progression. There is accumulating evidence that EMT confers migration, invasion, and metastatic capacity, and multidrug resistance to tumor cells [33, 34]. Therefore, we believe that fibrinogen may contribute to cell migration by inducing the EMT.

## Conclusion

Based on our findings, we suggest measuring the plasma fibrinogen levels preoperatively in new GBC patients to evaluate tumor progression and outcome. Furthermore, we conclude that fibrinogen enhances cell migration and invasion *in vitro*. However, our study was limited by its retrospective design and the small number of included patients. Thus, further studies of larger numbers of patients and prospective studies are required to confirm the present results.

## Abbreviations

GBC: Gallbladder cancer
PT: Prothrombin time
aPTT: Activated partial thromboplastin time
TT: Thrombin time
INR: International normalized ratio
ROC: Receiver operating characteristic curves analysis
OS: Overall survival
EMT: Epithelial-mesenchymal transitions
SEER: Surveillance, epidemiology, and end results
TNM: Tumor, node, metastasis
AJCC: American Joint Committee on Cancer
CBD: Common bile duct
qRT-PCR: Quantitative real-time PCR analysis
PPV: Positive predictive values
AUC: The area under the ROC curve
ALT: Glutamate pyruvate transaminase
AST: Glutamic oxalacetic transaminase
WBC: White blood cell
CEA: Carcino embryonic antigen.
